# Medical Societies and Clinical Reports—Philadelphia County Medical Society

**Published:** 1858-05

**Authors:** 


### SELECTIONS.

From the Medical and Surgical Reporter.

*Medical Societies and Clinical Reports.*

PHILADELPHIA COUNTY MEDICAL SOCIETY.

Feb. 10th, 1858. 7½ P. M. Dr. Mayberry, Vice President in the chair. While awaiting the arrival of Dr. T. F. Betton, the lecturer of the evening, Dr. Turnbull related a case of double cephalamatoma.—He was called to a lady in labor, which was so rapid, that the child was born when he arrived. At that time, he noticed nothing remarkable about the child. On the fifth day, his attention was called to a tumor on the head, in fact, two tumors, one on each parietal bone. They were about four and a half inches across. He directed a spirituous lotion, but they still inrceased, and about the seventh or tenth day, he could feel a peculiar hard ridge around the edge of the swelling. He consulted with Dr. J. F. Meigs, who mentioned the fact of having cured such a case, by poulticing and advised a like treatment in this instance. But though poulticed with much care every four or five hours, on the twentieth day there was no material diminution. He then inserted an exploring needle, and blood issued freely. It was evidently under the pericranium. The family being anxious for an operation, he made a free incision at the lower end of one tumor, and in consequence of the free effusion of blood, was compelled to use compresses, and cold applications. When this had ceased, he inserted patent lint, and applied adhesive straps, bandages, and a cap over all. On the following morning, he found the cap saturated with blood, and removed all the dressing, except the adhesive straps. Now, thirty-six days from the com mencement, the tumor has almost wholly disappeared. That on the other side, he did not open, but applied straps, and it also gradually disappeared.

Dr. Condie said these were rare occurrences, he believed, in this city. He had never met with a case; in a practice of fory-three years, where the blood, as in the case related this evening, was situated upon or beneath the pericranium. Tumors are common, in which the blood is effused between the laminæ of the scalp. It would be an interesting fact, could it be determined, to know whether, in Dr. Turnbull's case, the presentation had not been changed in the course of the labor. It is a very common opinion, and perhaps a correct one, that these tumors are produced by the pressure of the uterus upon the fœtal head during labor, the blood being driven into the most advanced part. The worst plan is to open them. If they are caused by a morbid condition of the pericranium, or of the vessels that penetrate the bones, the best plan is to keep them closed. Cold compresses are preferable in his opinion to warm poultices. In Europe, they apply astringent lotions, but he could think of none there, who recommended warm applications. These tumors have been, and are very liable to be mistaken for fractures of the bones.

Dr. Coates asked on what part of the parietal bones these were situated?

Dr. Turnbull replied that they were on the centre of the bones, directly over each boss, and at present, each bone seems somewhat enlarged.

Dr. Jewell had had three of these cases, and all after tedious labor: each was situated on the presenting part. The first remained over three weeks. He used cold astringent lotions, and not succeeding, followed these by poultices; had suppuration, opened the abscess, and extensive purulent discharge followed which left the child with a deformed scalp, and on this part the hair never grew.

In the second case, he used iodine lotions, and after ten or fifteen days, it began to subside, and was soon well. In the third, he also applied the iodine, for ten days, or two weeks and not succeeding, used warm bread and milk poultices, and absorption rapidly followed. The cupped appearance, to which Dr. T. alluded, was present in all these cases; it felt like a depression of the bone, but he did not consider it peculiar to the disease.

As the lecturer had not arrived, and it was past the time for expecting him, Dr. Condie proposed for the consideration of the members, an important subject, and one which may be made to throw considerable light on the pathology of disease, &c., viz., *intra-uterine diseases.*—Smallpox has been communicated to the fœtus, and it has been born with the eruption. Children have been born with extensive tuberculization of various organs, which, in some cases, had gone on to softening, and produced inflammation of the neighboring parts. Sometimes, we had disease, when the mother exhibited no symptom of it, as pneumonia, peritonitis, &c. Numerous cases are related of the latter disease, where adhesions have been formed, showing that inflammation of a severe grade had existed. Prof. Simpson, of Edinburgh, had published a paper in which he refers to a number of cases. In a large number of the instances on record, Dr. C. had found the mother had erysipelas, when the child had peritonitis. As the relation between erysipelas and peritonitis is now fully recognized by the profession, and there is not the least doubt but that erysipelas is a disease of the blood, the external phenomena, and concomitant affections of internal organs are merely results of the same blood disease. The mother had escaped, and the fœtus had had peritonitis with effusion and adhesions. The modern doctrine of phlebitis as a cause of pyemia, is that the phlebitis is the result of the diseased condition of the blood itself, and the diffused abscesses are not the result of pus taken into the circulation and deposited in particular parts, but that the depots met with in different organs in cases of empyema are in fact small abscesses produced by inflammation occurring at the place where the pus is discovered. The whole subject of intra-uterine diseases is but in its infancy, and curious results are no doubt to be derived from its close investigation.

One other form of disease is common in the fœtus in utero, and that is erysipelas; in the great epidemics of puerperal fever, at least two-thirds of the children were born with it upon them, and in many, there was peritoneal inflammation.

Dr. COATES mentioned a case of a child with cyanosis, which died in five months, where was found an open foramen ovale, and the lungs were crowded with tubercles, accompanied by emphysema of the kind ascribed by Rilliet and Barthez to children, with large cavities between the lobules, and some of these were one and a quarter inch long.

At the risk of producing disgust at the breakfast table, he would mention a phenomenon concerning eggs. Many were found on breaking them after boiling, to have the membrane adhering to the albumen so strongly, as to tear out patches of its substance. This was formerly rare, but at present a very large number of the eggs were so affected. He considered it due to an amnitis, or inflammation of the amnion, produced by carrying over railroads.

Dr. MAYBERRY had noticed several instances where children were not susceptible to the vaccine disease, where the mother had been vaccinated while carrying them, and had observed the same result from modified smallpox.

Dr. CONDIE remaked, that in the first of the modern epidemics of smallpox, in this city, a lady was affected with the modified form during the eighth month of pregnancy. It was light, and no remedy was required; but few imperfect pocks appeared. The child was born covered with genuine smallpox, and died within one hour. He never saw a more beautiful specimen of discrete smallpox, and some pocks had gone on to the period of maturation.

Dr. FLEMMING had attended a lady in labor, who was delivered of a child in a putrid condition. Scarlet fever had been in the family; one child had died with it, while the mother was far gone in her pregnancy. Since that time, she had not felt any movement of the fœtus. The presumption was that the child had died of scarlet fever.

Dr. JEWELL had, like Dr. Condie, a case of a young father who died of malignant smallpox on the seventh day. On that day, his wife was confined, having had varioloid previously, and the child was covered when born, with a full crop in the first stage, and died of the malignant form.

In connection with this, he mentioned a case of a child born with a mark similar to a vaccine mark on its arm; the mother had been vaccinated during pregnancy.

Dr. EMERSON, without intending to endorse this last, but considering it as a mere coincidence, would say, that the subject of vaccination during utero-gestation was of importance to decide. In epidemics, pregnant women were desirous of being revaccinated. Some medical men, of very good authority, decline so doing. He would like to hear the sentiments of the members on that point.

Dr. CONDIE asked what possible objection could exist, when we frequently vaccinate a child but a few days old. In the Foundling Hospitals of Paris, every child was vaccinated immediately on being received and distinguished accoucheurs vaccinate within the first three days.—The vaccination of the mother could not produce abortion, and if it really did extend its protective influence to the child, what could be the objection to its perforamance? What harm could possibly result?

Dr. R. K. SMITH remarked that the queston started this evening opened an interesting field of inquiry. The important practical point to discover, was the one alluded to by Dr. Emerson, viz: "the propriety of vaccinating or revaccinating pregnant woman." The remarks made by the chairman and Dr. Condie presented this subject to the mind in two different points of view. If revaccinating the mother protected the child in utero, or on the other hand, if, as Dr. Condie seemed to infer, it communicated true variola to the offspring, then, indeed, it was important that the question should be decided.

Dr CONDIE (interrupting). I said nothing of the kind.

Dr. SMITH. You certainly related a case that led to that inference, and so did Dr. Jewell.

Dr. JEWELL (interrupting). I said nothing about revaccination, or the vaccine disease producing smallpox in the child; I said where modified smallpox had been developed in the pregnant woman, it produced the true disease in the fœtus in the case above named.

Dr. SMITH. Well, if Dr. Jewell will explain how modified smallpox or variola vaccinia can occur without vaccination, I will give up the point. He certainly understood both the gentlemen to state their cases precisely as he had related them, and if he was mistaken in regard to Dr. Condie, he apologized for calling him up. He contended that if a child was born covered with a true variolous eruption, imparted to it by the mother from revaccination, it was an operation not to be thought of in pregnancy; but he maintained the diseases of variola and kinepox were entirely different, and would not produce each other. If the views of those gentlemen were correct, it seems the mother conveyed to the child a disease of which she had not the first symptom, as cowpox is a new disease. He did not rise to discuss the question, but simply to refer to it, as a very interesting one, and to suggest that it be made the subject for a future meeting, when gentlemen would come prepared to investigate it thoroughly.

Dr. CONDIE said that which proves too much, proves nothing. If Dr. Smith's ideas were correct, it would be unnecessary to resort to vaccination in a child born of a vaccinated or variolated female. The protection affoded by vaccination must result from a peculiar change wrought in the tissues of the various organs of the being by the vaccine virus.—We have a new being formed by, and in connection with the maternal organs, which had already undergone this change, in consequence of which she may have the disease in a modified form, or not at all, but the child whose tissues have not undergone the peculiar change, will have it in the unmodified form. Now all these diseases, smallpox, varioloid, and the vaccine disease, are the same in their *first elements.* In epidemics, a patient with varioloid will, and frequently does, give the genuine smallpox to others. It has been shown by experiments in Denmark, Holland, and Germany, that they are one and the same thing; and it has been proposed by Dr. Mohl, of Copenhagen, to inoculate the cow, in order to produce a fresh supply of vaccine matter; he says that he has produced vaccine matter in this way. There is no direct communication between the circulation of the mother and the child By microscopical examination of the blood we find none of its leading features to be identical in the mother and child. There is no connection; they are two independent beings; each builds up its own tissues.

Dr. R. K. Smith asked how the poison of disease was communicated to the fœtus by the mother if there was no participation by the fœtus in the circulation of the mother? If vaccine disease and variola are similar, why vaccination was resorted to in order to protect rather than inoculation?

Dr. Condie answered that it was easy of explanation. If we apply matter to the skin, or arsenic be placed in the food of the mother, it would poison the fœtus. The umbilical vessels take up this, to form blood for the fœtus, and if the blood of the mother is poisoned, the fœtus feels it. But not in all cases are diseases communicated to the fœtus.—These are the exceptions. The modification of the variolous matter which it undergoes by passing though the cow, renders it less dangerous. The vaccine disease is not contagious, but varioloid will give off exhalations and fomites, which poison others. Thousands of cases have proved that varioloid will produce genuine smallpox. That vaccine matter undergoes a change, is admitted by all the Europeans, with the exception of the English.

Dr. Emerson, in view of the acknowledged risk of a woman in gestation communicating disease to the fœtus, thought the doctrine, if it be true, that variola and vaccine disease are identical, would warrant us in making a distinction in regard to vaccinating or revaccinating a pregnant woman. Revaccination was justifiable, but the other was not, as it might produce smallpox in the fœtus.

Dr. Nebinger thought himself justified in concluding that children in utero do suffer and die from disease, and also that mothers in this condition have disease which are transmissible, and yet do not transmit them. At this point he would relate three instances:—

A lady died three days after her accouchement of malignant smallpox; the child is living still. Anxious to know the result of disease as to the offspring, he followed up the case, and found no evidence of ever being influenced by the variolous poison. Another lady, seven or eight months gone, had smallpox distinct, and she got safely through; the child was born, and is living; there is no evidence of smallpox having been produced on it. Fearing the poison that might still be lurking about the house, he vaccinated the child and it was fully affected by the vaccine disease. A little girl had violent smallpox. The mother was pregnant; two weeks rolled around, and the girl being convalescent, the mother was confined, and the child vaccinated the day after. This run its course regularly, and varioloid resulted. He did not intend this as an evidence that smallpox was not produced in utero, only as important to know the pros and cons, yeas and nays, of the subject. He did not conclude that cases could not occur of the fœtus receiving disease from the mother; on the contrary, from the well authenticated facts of children being born with the disease (smallpox for instance) that the mother had but a short time prior to delivery, he believed that disease could be conveyed to the fœtus; and the cases related by him were not offered to disprove the fact of the child in utero being affected by the disease the mother was suffering with, but were offered as evidence to prove that the child in the uterus, as is known to be the case out of the uterus, posseses that peculiar condition of system which makes it susceptible to morbific influences. Hence it is that if the mother whilst pregnant should be brought under the influence of the vaccine virus, there is no certainty that the fœtus would be influenced also.

Dr. COATES remarked, in regard to what Dr. Jewell had said conerning coincidence of *marks* on the fœtus, that when young, and engaged in the Pennsylvania Hospital, he saw a pregnant woman who had been badly burned, and the child was born with several patches of skin off the legs, remarkably like the burns. He believed that as the child was killed by the accident, putrefaction had caused this.

Dr. JEWELL saw an interesting case of the kind. A child fell in the street, and lacerated its ear; it was brought to the mother, in her third month of pregnancy, who applied a napkin to the part, took it off, and seeing the whole cheek covered with blood, was much alarmed. When the fœtus was born, the right side of the face was covered with a varicose aneurism. But, of course, this was merely a coincidence. He asked that Dr. Condie would give them some new theories to account for this.

Dr. CONDIE replied that it was a curious idea, the gentleman asking for theories, when in all his publications there would be found very little theory. An incident is related by an admirable author, Dr. Buchan, in his "Advice to Mothers." He gives a case, where the shock was so great that the child ought to have been deformed, if it was possible for the mind to effect it. A female in Edinburgh, six months gone, while sitting in her room was much startled by an escaped monkey jumping upon her shoulder. In great alarm she sent for Dr. B., sure that her child would be a monkey, or something horrible; but lo! the fright had no effect. In another case, a baker's wife, in her sixth month, had her husband brought home from a riot with his right shoulder injured.—When the child was born it had a nævus on the left shoulder. Now, as the nurses said this was a mistake, nature should have put it on the right shoulder. In looking over the last edition of Dr. Watson's work, he was much surprised to find that gentleman believing that the imagination of the mother may produce an effect on the fœtus. He could not see how, as there was no nervous connection.

Dr. CURTIS saw a lady in her first confinement, who, when the child was born, and before she had seen it, asked about the cherry. On enquiry, he learned that three months before, her brother had thrown a black cherry which struck her on the head, and she was certain the child would be marked with a cherry. On examination,he found a nævus exactly on the top of its head, very much resembling a black cherry.

Dr. COATES did not believe that the imagination of the parent could impress on the body of the child any mark resembling at all the object which had affected the imagination in a lively manner. But he could not consider this proved by the absence of nerves in the umbilical cord. Gentlemen much respected for their science, who where believers in animal magnetism, considered that the nervous influence could be projected from one individual into the body of another. The nerves end in loops or direct terminations; with considerable spaces between them, as demonstrated by the microscope, and yet we have the nervous action going on without impediment from that cause.

Dr. EMERSON remembered a case of a child born with spina bifida.—The mother gave, as she thought, a clear account of its cause. She had been much frightened by a great danger to which one of her children had been subjected, in the early part of her pregnancy. The child had been struck with an axe on the lower part of the back, and when she heard it, much shocked she clapped her hand to that part of her back mechanically, and firmly believed this was the cause of the spina bifida. He regarded it merely as a coincidence.

Dr. CONDIE thought that a very stupid archeus governed in the case related, not to put the hand on the part also.

If it were not so late, he would like much to argue some facts stated by Dr. Coates concerning the nervous system but hoped to have an opportunity to do so at some other time.

Adjourned.

